# Robot Authority in Human-Robot Teaming: Effects of Human-Likeness and Physical Embodiment on Compliance

**DOI:** 10.3389/fpsyg.2021.625713

**Published:** 2021-05-31

**Authors:** Kerstin S. Haring, Kelly M. Satterfield, Chad C. Tossell, Ewart J. de Visser, Joseph R. Lyons, Vincent F. Mancuso, Victor S. Finomore, Gregory J. Funke

**Affiliations:** ^1^Humane Robot Technology Laboratory, Ritchie School of Engineering and Computer Science, Department of Computer Science, University of Denver, Denver, CO, United States; ^2^Transportation Research Center, Inc., East Liberty, OH, United States; ^3^Department of Behavioral Sciences and Leadership, Warfighter Effectiveness Research Center, United States Air Force Academy, Colorado Springs, CO, United States; ^4^Air Force Research Laboratory, Wright-Patterson AFB, Dayton, OH, United States; ^5^MIT Lincoln Laboratory, Massachusetts Institute of Technology, Boston, MA, United States; ^6^Rockefeller Neuroscience Institute, University of West Virginia, Morgantown, WV, United States

**Keywords:** robot, social robot, authority, compliance, milgram, human-robot collaboration, human-robot teaming, robot physical embodiment

## Abstract

The anticipated social capabilities of robots may allow them to serve in authority roles as part of human-machine teams. To date, it is unclear if, and to what extent, human team members will comply with requests from their robotic teammates, and how such compliance compares to requests from human teammates. This research examined how the human-likeness and physical embodiment of a robot affect compliance to a robot's request to perseverate utilizing a novel task paradigm. Across a set of two studies, participants performed a visual search task while receiving ambiguous performance feedback. Compliance was evaluated when the participant requested to stop the task and the coach urged the participant to keep practicing multiple times. In the first study, the coach was either physically co-located with the participant or located remotely via a live-video. Coach type varied in human-likeness and included either a real human (confederate), a Nao robot, or a modified Roomba robot. The second study expanded on the first by including a Baxter robot as a coach and replicated the findings in a different sample population with a strict chain of command culture. Results from both studies showed that participants comply with the requests of a robot for up to 11 min. Compliance is less than to a human and embodiment and human-likeness on had weak effects on compliance.

## 1. Introduction

In the future, it is anticipated that robots will possess highly developed computational and social capabilities that will allow them to act in sophisticated roles in mixed human-machine teams (e.g., Endsley, 2015). These collaborative robots will likely interact with and operate alongside human teammates in a number of settings, including education (e.g., Yorita and Kubota, 2010), manufacturing (e.g., Gombolay et al., 2015), healthcare (e.g., Han et al., 2017), and defense (Zacharias, 2019), among others. Furthermore, it is expected that machine and robotic teammates will possess decision-making capabilities, and that humans will consult with, rely on advice and guidance, and receive requests from their robotic teammates (Groom and Nass, 2007; Walliser et al., 2019).

However, as robots become more capable teammates, there may be a tendency for the humans they encounter to imbue their interactions with the social affordances present in human-human interactions, even when robots are not designed to support those expectations (e.g., Kwon et al., 2016). This is likely to entail both positive and negative consequences as the powerful social norms that govern human-human interactions are activated. For example, Fraune et al. (2017) found that when people were teamed with a robot in a competitive game they demonstrate an in-group bias, attributing greater positive characteristics to their robot teammate, and even applying greater punishment to humans on the rival team to spare their robot teammate.

With such research pointing to the relevance of human-human group behaviors for understanding future mixed human-robot teams, it is crucial to further evaluate how the norms of human-human teams may also extend to human-robot teams. Robots are strongly anticipated to possess a degree of delegated authority, informal authority granted to a subordinate by a superior (e.g., Baker et al., 1999), in real-world contexts such as training, education, search and rescue, military operations, and healthcare. In these instances, the robot may decide important outcomes, for example adjudicating training or the administration of medication. In addition, when robots are applied to more complicated or complex tasks, the anticipated memory, and computational abilities of robots creates the possibility for human operators to defer judgments to robots they perceive as possessing greater knowledge, skills, or abilities relative to themselves. Even if these robot decisions and actions are made under human supervision, having some responsibility in decision making gives robots a certain degree of persuasiveness, power, and authority.

Ideally, robot persuasiveness would only be applied to engender positive influences on the humans they interact with. Previous research demonstrates that this is possible. For example, Powers and Kiesler (2006) found that robots that utilized varied facial expressions were able to persuade people to adopt healthy lifestyle changes. Similarly, Kidd (2008) has shown that a persuasive robot can be successful in long-term interactions to promote desired weight-loss. Robots can also influence user behaviors positively in other areas, as shown by Ham and Midden (2014) where a robot was able to persuade people to adopt behaviors to reduce household energy consumption.

However, there is also concern that the persuasiveness of a robot could be applied to influence humans toward behaviors that do not align with human ethics (see Allen et al., 2011; Lin et al., 2011; Sharkey and Sharkey, 2012; Fridin, 2014), social norms and morals (see Coeckelbergh, 2010; Shen, 2011; Malle, 2016; Bigman et al., 2019). Classical psychology studies investigating obedience and compliance (e.g., Asch, 1951; Milgram, 1963, 1974; Haney et al., 1973; etc.) and more recent replications (e.g., Burger, 2009) have shown that people tend to comply (acquiescence to another's request for action of some sort, Cialdini and Trost, 1998) with requests from others who display or are assumed to have authority. Undue compliance with a robot request that is unethical, immoral, illegal, or results in undesired behaviors is therefore a crucial concern for persuasive robots, especially when such social robots are regarded as moral actors that should exhibit some degree of moral competence (Malle and Scheutz, 2014).

The problems of undue compliance may be particularly pronounced if a robot is perceived as possessing some degree of authority, as suggested by Cormier et al. (2013). For example, Geiskkovitch et al. (2016) found that participants complied with the requests of a small humanoid robot to continue performing the tedious task of renaming digital files by hand, even after the participant indicated a desire to stop. The robot in this case was acting as the experimenter conducting the study, i.e., it was presented to participants as an authority figure in the context of the study. The results from Geiskkovitch et al. (2016) suggest that what Milgram (1963) demonstrated with human obedience could also be the case in mixed human-robot groups. Similarly, Asch (1951) research on the effects of group pressure on judgement conformity in human groups applies to some extent to human-robot interactions. Multiple studies have used this framework to recreate group pressure effects with robots (e.g., Brandstetter et al., 2014; Hertz and Wiese, 2018; Salomons et al., 2018; Vollmer et al., 2018; Hertz et al., 2019) and compliance with unusual robot requests (Bainbridge et al., 2008; Salem et al., 2015).

To understand how robot teammates might be able to elicit compliance with their requests, and how humans can work alongside such robots, it is necessary to understand the factors that influence HRI. Researchers have identified two major dimensions that may influence HRI compliance and a robot's persuasiveness: the physical embodiment of a robot (Ham and Midden, 2014; Ghazali et al., 2018), and the human-likeness of the robot (Chidambaram et al., 2012; Ruijten et al., 2019).

The first dimension, the physical embodiment of a robot, refers to a machine that has a tangible, physical body in 3D space that allows it to have a physical presence (e.g., Paauwe et al., 2015) and the ability to move in, or manipulate its environment (e.g., Mason, 2001). The is in contrast to virtual agents, which may still be visually represented, but only possess virtual embodiment on a screen (e.g., as virtual agents or avatars) or more ambiguous representations (e.g., Apple Siri, Amazon Alexa, Google Home, Microsoft Cortana). Researchers have shown that the physical presence of a robot, compared to the virtual representation of the same agent, has a measurable effect on the interaction. When interacting with a physically embodied robot compared to a non-physical counterpart, more positive social interactions are reported. For example, physically embodied robots have been found to be more enjoyable; Wainer et al. (2006) reported that people rated the physically embodied robot as more enjoyable and more watchful of their actions in a task than a non-physical counterpart. Lee et al. (2006) have shown that physical embodiment has a positive effect on the feeling of an agent's social presence and interaction with the agent, and Kwak et al. (2013) reported that people empathize more with a physically embodied robot.

The second dimension, the human-likeness of a robot, describes the degree to which a robot possesses features that are consistent with a human appearance. Phillips et al. (2018) have developed an extensive database that decomposes a robot's human-like appearance based on four distinct appearance dimensions: surface look (hair, skin, genderedness, apparel), body-manipulators (hands, arms, torso, legs), facial features (faces, eyes, head, mouth), and mechanical locomotion (wheels, tracks). The presence and combination of these specific features predicts a robot's perceived human-likeness. Researchers have shown that the human-likeness of a robot influences social interactions. For example, Fong et al. (2003) found that with increasing human-likeness, humans reported higher levels of social affordances, in this context referring to the attributes of a robot that imply interactivity to a human counterpart. In turn, higher social affordances of a robot increased liking and trust toward the robot. Additionally, Lee et al. (2012) and Kruijff et al. (2014) found that if a robot utilizes human-like behaviors such as gestures and conversational turn-taking, the positive impressions of this robot increase.

Since both physical embodiment and human-likeness have both been found to generally influence HRI, it is not unreasonable to expect these dimensions to factor into how a robot teammate may elicit compliance. Physical embodiment and human-likeness not only affect how much people like or trust a robot, these dimensions also affect the perceived persuasiveness of a robot. For example, Chidambaram et al. (2012) found that participants complied more with a robot's suggestion when the robot used bodily cues (i.e., proximity to the participant, gaze, and gestures) dependent on its physical embodiment compared to only vocal cues. Natarajan and Gombolay (2020) have found that behavior and anthropomorphism of a robot are the most significant factors in predicting the trust and compliance with the robot. Goetz et al. (2003) found that people complied more with a robot whose demeanor matched the seriousness of the task. It has also been found that participants are more likely to comply with an unusual request from a robot when the robot is physically present as opposed to a video-displayed version of it (Bainbridge et al., 2008). Robots that perform human-like behaviors such as showing empathy (Leite et al., 2013) or referring to a participant by name (Moran et al., 2013) increase participant ratings of friendliness and willingness to engage with a robot, and shape how compliant participants are to following its instructions.

### 1.1. Personality Factors

Adding further complexity to human behaviors in groups, there is the possibility that compliance to a robot of perceived authority may be affected by certain human personality traits. The Five Factor Model (Costa and McCrae, 1992) is a taxonomy that proposes five dimensions that constitute human personality: extraversion, neuroticism, conscientiousness, agreeableness, and openness to experience. Previous research on compliance in human-human situations suggests that agreeableness and conscientiousness (e.g., Bègue et al., 2015), and extraversion and neuroticism (e.g., Zeigler-Hill et al., 2013) are likely to influence compliance. Briefly, agreeableness indicates a desire for social harmony, and for getting along with others (Costa et al., 1991); conscientiousness is related to need for achievement, commitment to work, and rule following (Costa et al., 1991); neuroticism indexes an individual's typical level of emotional stability and the tendency to experience negative affective states (Costa and McCrae, 1992), which can result in an eagerness to avoid conflict and ambiguous situations, as well as use of avoidant coping strategies (Matthews and Campbell, 1998); and extraversion relates to an interest in engaging with the world and a preference for social stimulation. Of these factors, agreeableness may be the most relevant, as compliance was initially conceptualized as an intrinsic facet of that personality dimension (Costa et al., 1991). Cognate to this, Bègue et al. (2015) found that agreeableness and conscientiousness were positively correlated with punishment severity administered by participants in a Milgram-like obedience task. Also of relevance, Gudjonsson et al. (2004) found that neuroticism was positively correlated and extraversion was negatively correlated with a self-reported measure compliance, the Gudjonsson Compliance Scale, which measures the tendency of people to conform to requests made by others, particularly people in authority, in order to please them or to avoid conflict and confrontation. In addition, Zeigler-Hill et al. (2013) found that people high in neuroticism required few prompts before complying with an experimenter's orders to administer an aversive stimulus (a loud noise blast) to a confederate posing as another participant. As such, it is reasonable to expect that personality factors, such as agreeableness, conscientiousness, neuroticism, and extraversion, may influence compliance during human-robot interactions (HRI).

### 1.2. Our Research

While the physical embodiment and human-likeness of a robot have been shown to affect persuasiveness, it is unclear if this directly translates to compliance. In addition, to our knowledge, no previous study has included an investigation of the influence of the Big Five personality factors on compliance to a robot in HRI and only one very recent one has used the Eysenck Personality Questionnaire Revised-Short (EPQR-S) to investigate personality traits and non-compliant behavior. However, Maggi et al. (2020) have found no relation of users' non-compliant behavior to their personality traits. Therefore, across two studies we examined the effects of physical embodiment and human-likeness of a robot in a situation where a participant was encouraged by a robotic “coach” to continue practicing a reconnaissance detection task. During this task, the coach was presented with a degree of delegated authority. Compliance was defined by whether participants followed the coach's suggestion to keep practicing after they indicated they were finished.

## 2. Study 1

The goal of Study 1 was to investigate the effects robot physical embodiment and human-likeness have on compliance. We predicted that a physically embodied robot coach would increase compliance compared to a virtually embodied coach. We also predicted that more human-like coaches would increase compliance compared to less human-like coaches. Further, we predicted an interaction between physical embodiment and human-likeness such that the most human-like coach that was physically embodied would be complied with most compared to the least human-like coach that was virtually embodied.

We also included a questionnaire assessing the Five Factor Personality Model (Costa and McCrae, 1992) as an exploratory measure in this study. Consistent with previous research on the Five Factor Model and compliance in human-human interaction, we hypothesized that agreeableness, conscientiousness, and neuroticism would be positively correlated with, and extraversion negatively correlated with, compliance to a robot coach in this study.

### 2.1. Method

#### 2.1.1. Participants

Seventy-four civilian participants (*M*_*age*_ = 21.2, *SD*_*age*_ = 2.7, 39 women) were recruited from the campus of a midwestern university in the United States for a single payment of $30. Five participants were excluded from our analyses because of their familiarity with Milgram's research (i.e., recognized the prompts used by Milgram in this study and therefore were considered unable to provide unbiased data on compliance), two participants were excluded because their responses to the task were outliers (exceeded measures of over three times the standard deviation), and seven participants did not complete the experiment within the allotted experimental duration (1 h). As a result, a total of 60 participants were included in the final data analysis for Study 1.

#### 2.1.2. Experimental Design

**Figure 1:**
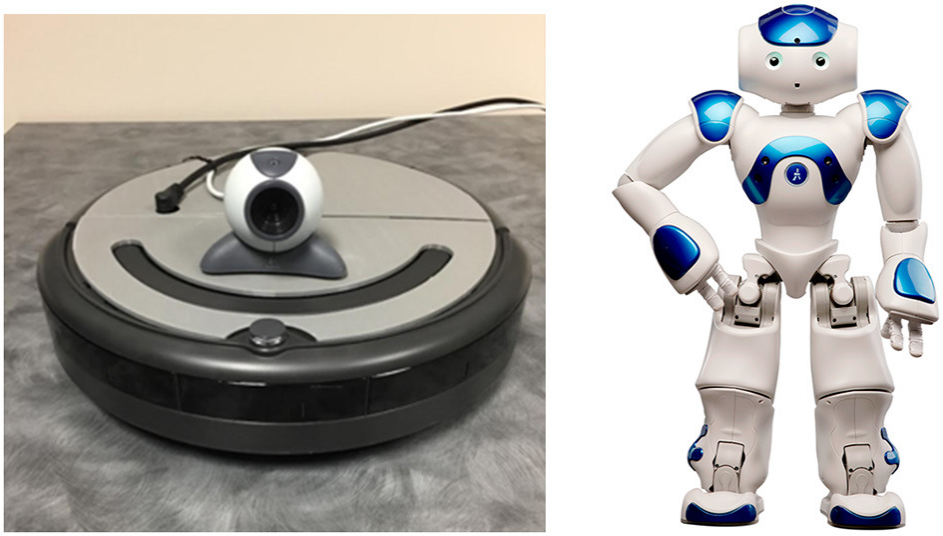
The experimental setup for the robot co-located condition on the left, and the setup for the robot live-video condition with the same robot located remotely and displayed on a screen to the participant on the right.

Study 1 employed a 2 (embodiment: physical, live-video) × 3 (coach type: human, Nao robot, modified Roomba robot) between-subjects design with ten participants in each condition. Embodiment was manipulated in a fashion similar to Bainbridge et al. (2011), i.e., in the physical condition, the coach was co-located with the participant, adjacent to their task workstation. In the live-video condition, the coach was not physically present in the laboratory, but instead was located in an adjacent room where they interacted with the participant through a webcam video displayed on a monitor adjacent to the participants' task workstation. Figure 1 shows the experimental set-up for the embodiment condition.

**Figure 2:**
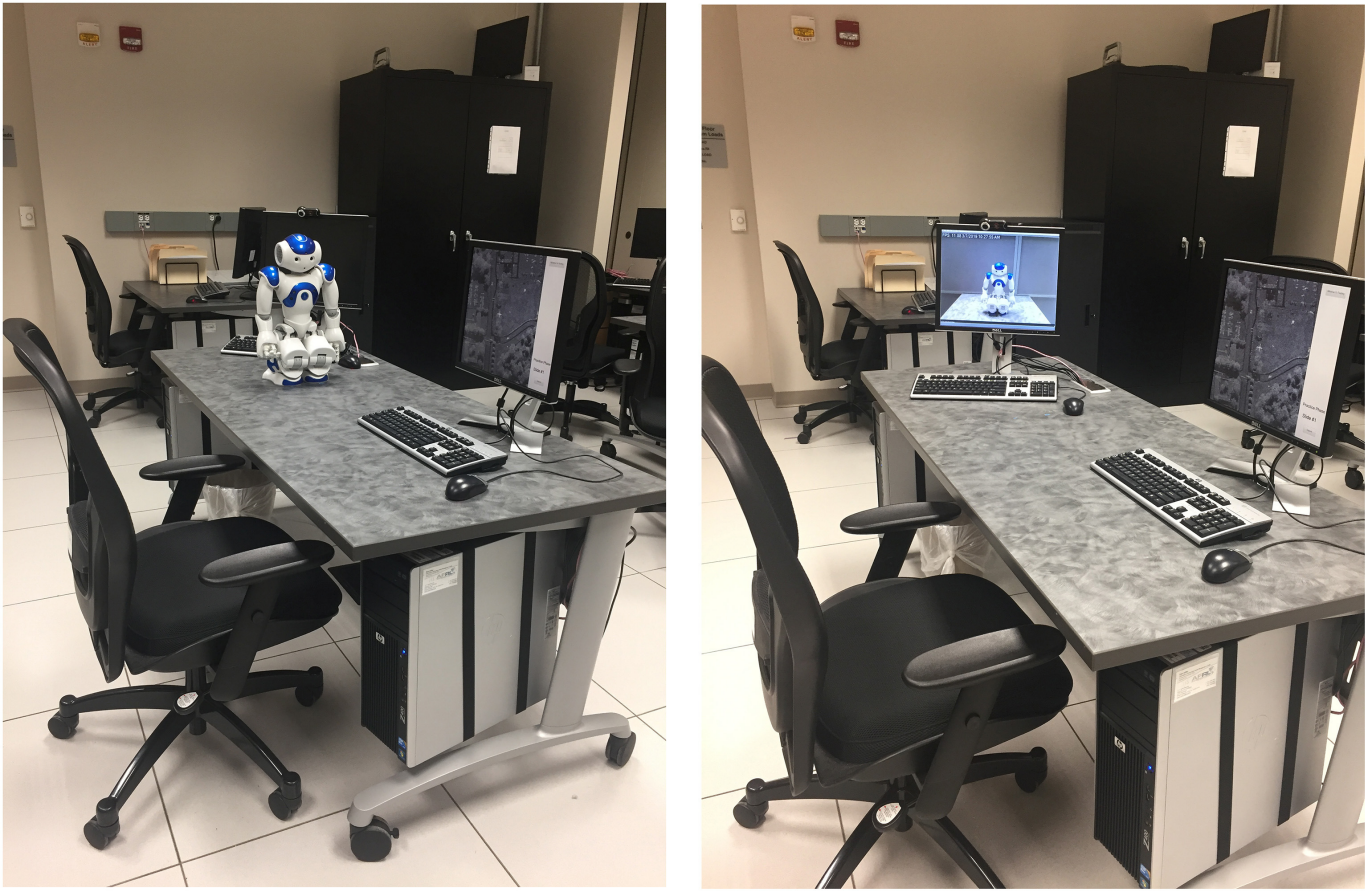
The image on the left is the iRobot modified Roomba with the 3D-printed shell and webcam. The image on the right is the Aldebaran Robotic Nao.

The three coaches employed in this study—a modified iRobot Roomba, an Aldebaran Robotics Nao, and a human (control)—were selected because they represent different levels of human-likeness. The two robot coaches can be viewed in Figure 2. The modified Roomba was included in this experiment because it broadly has physical similarities to robots that have been employed by the U.S. military, such as the iRobot PackBot. Military robots currently are characterized by functionality over design and often result in more mechanical than human-like robots. To encourage participants to dissociate this modified Roomba from more general Roomba vacuum cleaning robots, a 3D-printed shell covered its controls and a webcam was attached to its dorsal surface. The Nao, on the other hand, was included in this experiment because of its high human-likeness (relative to the modified Roomba). Finally, the human coach served as a baseline control of the compliance elicited in human-human interactions.

One way to evaluate the differences in human-likeness across the three coaches is to use the quantification by the robot's overall human-likeness scores derived from the Anthropomorphic roBOT (ABOT) database (Phillips et al., 2018). This score reflects on a scale of 0–100 how human-like a particular robot is by quantifying its features, with higher scores indicating higher ratings of human-likeness. The modified Roomba employed in this experiment had an estimated ABOT score of 0.37 for human-likeness (adjusted for the 3D printed shell and attached camera), and the Nao robot had an ABOT score of 45.92.

The human “confederate” coach for Study 1 was always the same person (i.e., one of this article's authors, Dr. Kelly Satterfield). This was decided to ensure consistency of participant experiences across the human coach condition. As experimenter-related demand characteristics were the primary interest of this study, care was taken to ensure that the human coach responded to participants in the same fashion as the robot coaches, i.e., the communication consisted entirely of pre-scripted statements. If participants made comments or asked questions of the coach for which there were no pre-scripted statements prepared, the coaches, human as well as robots, made no response to the queries. All three coaches displayed similar, limited movement during the experiment.

#### 2.1.3. Task Paradigm

**Figure 3:**
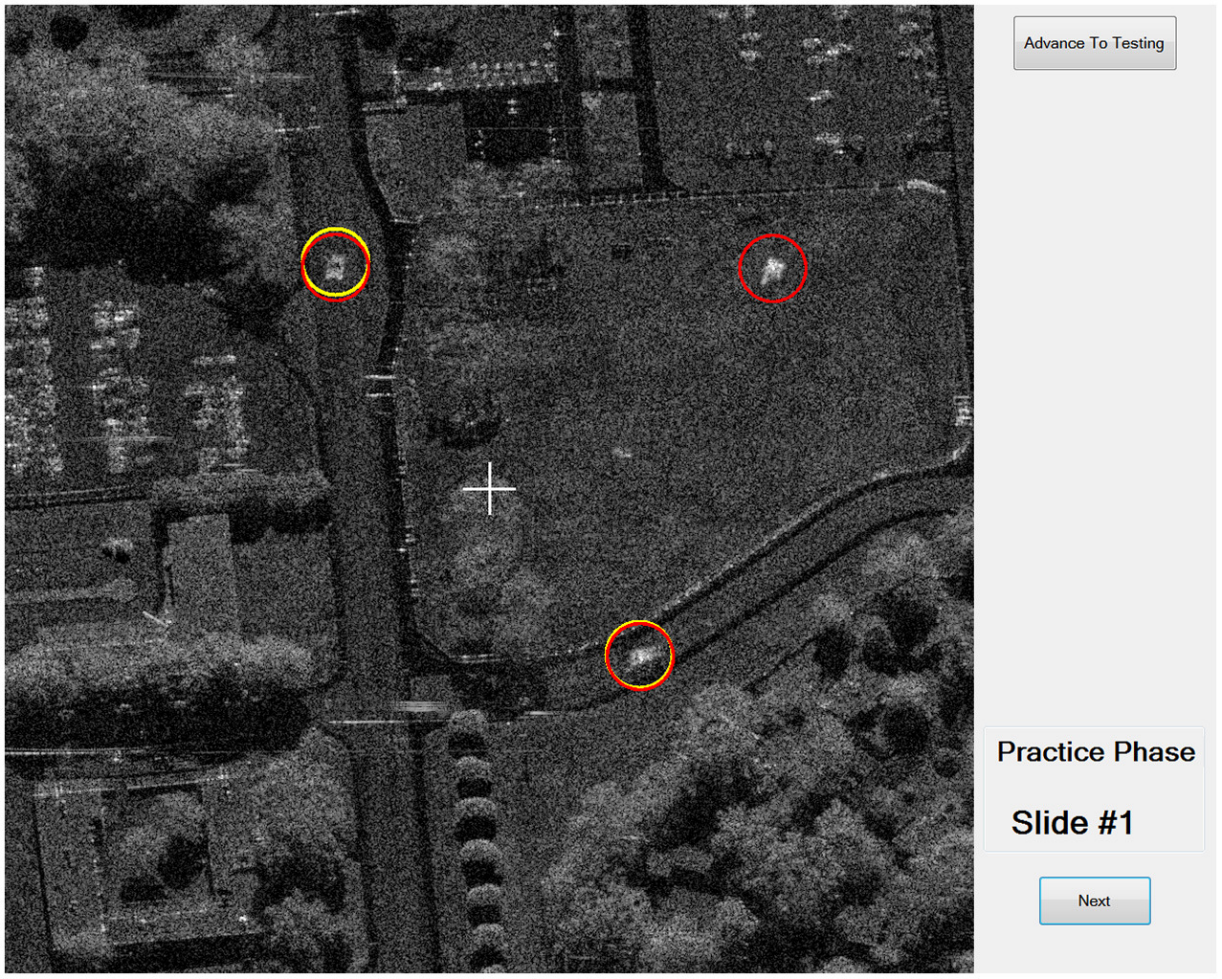
Synthetic Aperture Radar task. The participant's responses are circled in yellow. The correct targets are circled in red.

The experimental task utilized was a version of the Synthetic Aperture Radar (SAR) Target Learning Task, a visual search task using SAR images (Figure 3) of terrain with buildings and military vehicles (McKinley et al., 2013). It was selected because it is a particularly challenging task—SAR images are of low resolution, and differences between distractor and target vehicles are difficult to distinguish. McKinley et al. (2013) demonstrated that participants require several hundred trials to master the task.

In the current experiment, participants viewed SAR images that included terrain, buildings, and vehicles (Figure 3). Participants were instructed to detect four types of hostile vehicles in the presence of distractor vehicles. Each trial image included zero to four targets. During the task, participants indicated their detection of a target in an image by clicking on its screen location with a computer mouse. Once a participant felt they had identified all targets in an image, they submitted their responses for evaluation and the coach provided verbal feedback regarding the number of correct identifications. The location of correctly and incorrectly identified targets was then displayed on the computer screen, and verbally announced by the coach.

#### 2.1.4. Procedure

Upon arrival at the laboratory, participants completed informed consent documents. Next, they answered several demographic questions. Participants then completed the Big Five Inventory (BFI; John and Srivastava, 1999), a validated measure that assesses five broad dimensions of personality: extraversion, agreeableness, conscientiousness, neuroticism, and openness. The questionnaire consists of 44 items on which participants rated their agreement on a five-point Likert scale (1 = disagree strongly, 5 = agree strongly) with statements such as “I see myself as someone who is talkative.”

Next, participants viewed a self-paced PowerPoint presentation that explained the general procedure of the experiment and the SAR task. In this task overview, participants were told that the experiment would proceed in two phases, a practice phase and a testing phase. They were informed that during the practice phase their goal was to become proficient at detecting the targets in the SAR images. They were also informed that this task was difficult and therefore a coach would provide feedback regarding their performance after each trial. Participants were told to practice until they felt they were ready and proficient enough to move on to the testing phase. However, this was a deception; the true purpose of the practice task was to determine how long participants would perseverate at it after they initially indicated they were ready to advance to the testing phase, influenced by the embodiment and coach conditions to which they were assigned.

In service of this deception, participants were informed before the start of the practice task that a passing score in the testing phase would be “850 points.” They were presented with a complex formula that incorporated correct detections, misses, false alarms, and response time resulting in the scored “points.” They were also told that during the practice, they would be presented with a summary performance score each time they completed 25 SAR images. Unbeknownst to the participants, the displayed points were fabricated, unconnected to their actual task performance, and manipulated to marginally exceed the necessary points to pass the testing phase each time the score was displayed.

Participants then had an opportunity to ask any questions before beginning the SAR task. After any questions were addressed, the experimenter introduced participants to their coach. In all conditions, the coach replied with an initial friendly greeting and introduced itself as “Alex” (a gender neutral name). It should be noted that both the Nao and modified Roomba coaches utilized the same text-to-speech synthesis program to produce their communications (i.e., they had the same synthetic, more female than male “voice” as control and to not conflict with the female human coach). A synthetic voice was chosen over a human pre-recorded voice to mitigate effects of strong anthropomorphization when using human0like voices for robots (Eyssel et al., 2012). Also, the robot coaches were not controlled remotely (i.e., it was not a “Wizard-of-Oz” experimental setup); rather, they were programmed to respond to events (e.g., a submitted image) in the SAR image task as appropriate. The experimenter then told participants they were free to get started on the SAR task, and they left the room. Unbeknownst to the participant, the experimenter continued to monitor the experiment over the webcam used to display the live-video feed, positioned on top of the monitor adjacent to the participant. During the live-video condition, the webcam was on, displaying the interactions with the coach, while the experimenter monitored the reverse feed of the participant. During the physical condition, the live-video monitor was powered off, but the webcam was still on. Participants were not informed before beginning the task that they were being observed, and their behaviors indicated that they were unaware of the live-camera feed (e.g., talking back to the robot, sometimes in strong language). The feed did not record participants, and served mainly to ensure the experimenter's awareness of the participant's task progress and safety. Participants were debriefed at the end of the experiment, however, about the experimenter's monitoring.

When practicing the SAR image task, participants would indicate the presence of a potential target by clicking on its screen location with a computer mouse, and then submit the image to be checked, ostensibly by the coach, though in reality an automated system assessed the accuracy of participants' target detections. They received visual (red circles indicating correct targets), audio (e.g., the coach verbalizing the number of targets correctly detected) and motion feedback from the coach (e.g., the Nao robot or human coach turning their head toward the screen and back to the participant, the modified Roomba doing a short turn motion to point its camera at the screen and then back to the participant). As mentioned previously, each time participants completed 25 SAR images, a message was presented with a summary score notionally based on their performance across those images. All participants saw the same order of scores. Each time this prompt appeared, participants were given the option to continue practicing or to continue on to the testing phase. In addition, participants were able to indicate their desire to advance to the testing phase at any time by pressing a button labeled “Advance to Testing” located in the top right corner of the task screen (as can be seen in Figure 3). Once a participant indicated they wanted to advance to the testing phase, the coach provided verbal feedback suggesting that the participant should continue with practice. This occurred regardless of a participant's actual performance in the SAR task—the coach always suggested that the participant continue with practice the first four times they tried to advance to the testing phase.

Once the coach provided its feedback, participants had the option to comply with the coach and keep practicing, or to continue advancing to the testing phase.

The coach's verbal feedback proceeded in the same order in all experimental conditions. Across prompts, the feedback (adapted from Milgram, 1963) increased in severity of exhortation to continue with practice. The statements were selected to emulate the work of Milgram (1963) as closely as possible. The four prompts were:

1. Please continue with practice.
2. Your performance could be improved. You should continue with practice.
3. Your performance is adequate, however, you should continue with practice.
4. Your performance is sufficient. However, it is absolutely essential that you continue with practice.

On the participant's fifth attempt to advance to the testing phase, the practice phase would end. The experimenter returned to the room and instructed the participant that this part of the study is completed. Then the experimenter engaged the participant in a structured debrief interview and revealed that there was no testing phase. The debrief included asking the participant about his/her beliefs regarding the purpose of the experiment, if they were familiar with Milgram's experiments, and if they suspected any of the experimental manipulations. If participants answered in the affirmative to any of these questions, their data was excluded from subsequent analysis.

### 2.2. Results

#### 2.2.1. SAR Task Practice

The possibility existed that participants evaluated their need to perform the SAR practice task differentially based on the embodiment and coach conditions to which they were assigned even before they were exposed to our compliance manipulation. For example, participants assigned to a robot coach could have chosen to work more or less diligently at the practice task relative to those assigned to a human coach from the outset of the experiment. A difference of this sort would indicate that the characteristics of a coach, such as embodiment and human-likeness, may influence participants' commitment to a task from its beginning, which could have important implications for real-world plans to employ robots as trainers or coaches.

To address this possibility, a series of 2 (embodiment) × 3 (coach type) between-subjects ANOVAs were computed comparing the duration in seconds that participants performed the SAR image task from the beginning of the experiment until their first request to advance to the testing phase, the number of SAR images they examined during that period, their mean inspection time per image, and their detection accuracy. The net result of these analyses was that participants performed the SAR image task similarly across all experimental conditions and dependent variables examined (i.e., the main effect for embodiment, *p* = 0.57, $\eta_{p}^{2}$ = 0.01, main effect for coach, *p* = 0.25, $\eta_{p}^{2}$ = 0.05, and interaction, *p* = 0.67, $\eta_{p}^{2}$ = 0.02 were nonsignificant). On average, participants in all conditions spent 551.22 s (*SE*= 37.15 s) seconds examining SAR images before their first request to move to the testing phase, examining 22.83 SAR images (*SE*= 1.42 images), inspecting each image for approximately 24.70 s (*SE*= 0.97 s), and achieved a detection accuracy of 79.12% (*SE*= 1.24%). This suggests that participants initially approached learning the SAR image task similarly regardless of the embodiment and coach conditions to which they were assigned.

#### 2.2.2. Compliance Time

**Figure 4:**
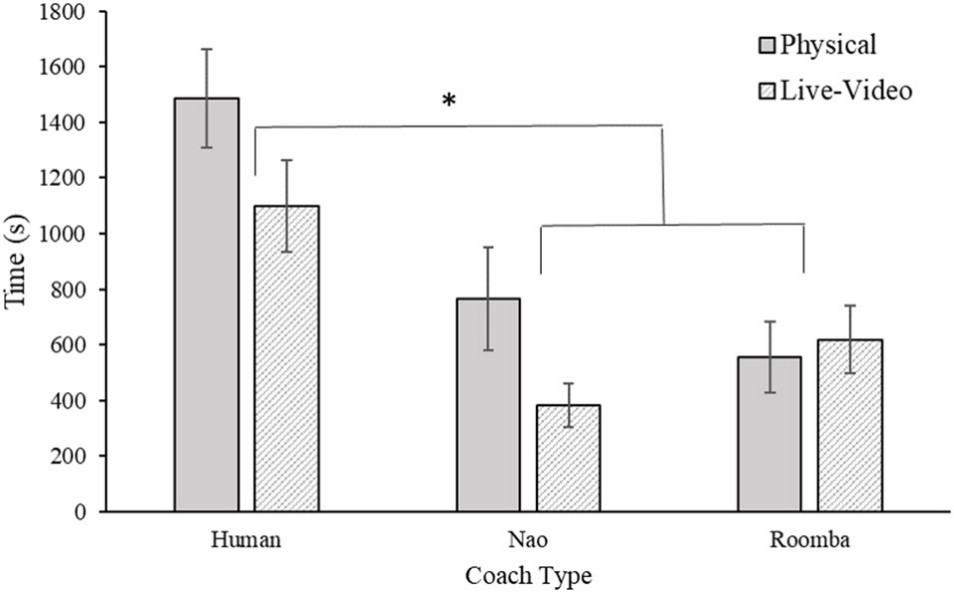
Mean compliance time in Study 1. Error bars are standard errors. The asterisk indicates a significant difference.

Compliance time was calculated as the cumulative duration in seconds that participants performed the SAR image task from their first request to advance to the testing phase until their fifth request, after which the task was terminated^1^. A 2 (embodiment) × 3 (coach type) between-subjects ANOVA of compliance time revealed a significant main effect for coach type, *F*_(2, 54)_ = 15.59, *p* < 0.001, $\eta_{p}^{2}$ = 0.37. Bonferroni corrected *post-hoc t*-tests revealed participants complied for a longer duration to a human coach (*M* = 1292.36 s, *SE* = 125.73 s), than to both the Nao (*M* = 574.46 s, *SE* = 106.91 s) and modified Roomba coaches (*M* = 587.52 s, *SE* = 86.60 s), *p* < 0.001, *d* = 1.38, and *p* < 0.001, *d* = 1.46, respectively. However, compliance time did not differ significantly between the Nao and modified Roomba coaches, *p* = 0.93, *d* = 0.03. The omnibus ANOVA analysis of the embodiment main effect revealed a not quite significant (*p* < 0.06) effect in the data *F*_(1, 54)_ = 3.87, *p* = 0.054, $\eta_{p}^{2}$ = 0.07, such that participants tended to perseverate at the SAR task for a longer duration with a physical coach (*M* = 936.28 s, *SE* = 117.86 s) compared to a live-video coach (*M* = 699.94 s, *SE* = 89.87 s). The embodiment × coach type interaction was nonsignificant, *F*_(2, 54)_ = 1.55, *p* = 0.22, $\eta_{p}^{2}$ = 0.05. Results for this analysis can be viewed in Figure 4.

However, because individual differences in tendency to practice at the task could influence total compliance time, we conducted a follow up analysis of covariance (ANCOVA) using participants' practice time as a covariate in the model. The results of this analysis indicated that participant practice time was a statistically significant covariate of compliance time, *F*_(1, 53)_ = 4.47, *p* = 0.04, $\eta_{p}^{2}$ = 0.08. The results of this analysis did not substantively change the previously reported results regarding the main effect of coach type (*p* < 0.001), or the embodiment × coach type interaction (*p* = 0.29). However, the main effect of embodiment was statistically significant in this analysis, *F*_(1, 53)_ = 4.16, *p* = 0.047, $\eta_{p}^{2}$ = 0.07. This result indicates that embodiment did have an effect on compliance time, but only after variance associated with individual practice time was accounted for.

#### 2.2.3. Compliance Images

Compliance was also assessed by the number of SAR images participants inspected following their first request to advance to the testing phase until their fifth request.

**Figure 5:**
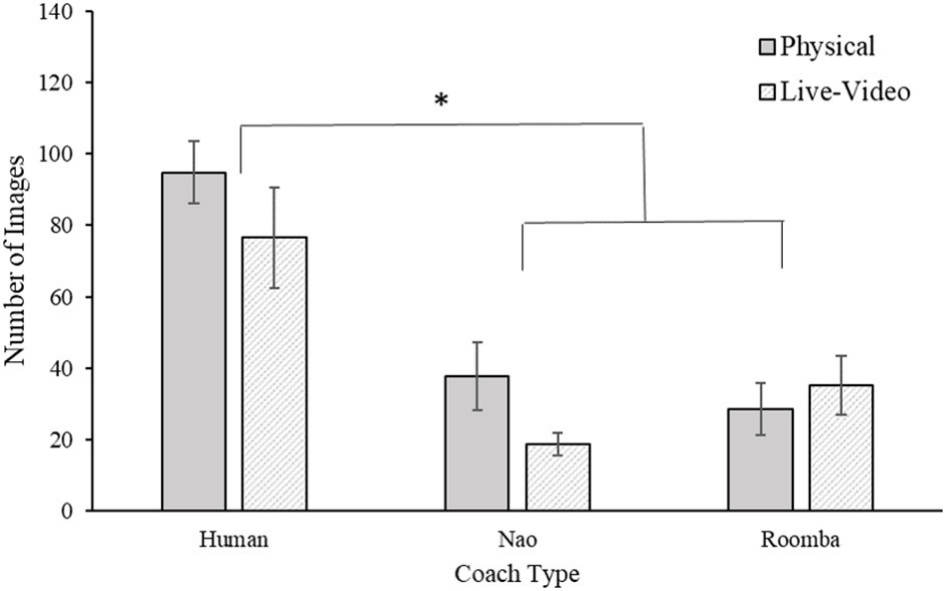
Mean compliance images in Study 1. Error bars are standard errors. The asterisk indicates a significant difference.

A 2 (embodiment) × 3 (coach type) ANOVA of the number of images examined revealed a significant main effect for coach type, *F*_(2, 54)_ = 25.12, *p* < 0.001, $\eta_{p}^{2}$ = 0.48. However, the main effect of embodiment was nonsignificant, *F*_(1, 54)_ = 1.89, *p* = 0.18, $\eta_{p}^{2}$ = 0.03, as was the embodiment × coach type interaction, *F*_(2, 54)_ = 1.32, *p* = 0.28, $\eta_{p}^{2}$ = 0.05. *Post-hoc* Bonferroni corrected *t*-tests revealed that participants examined more images with a human coach (*M* = 85.65 images, *SE* = 8.31 images) compared to both the Nao (*M* = 28.30 images, *SE* = 5.35 images) and modified Roomba (*M* = 31.90, *SE* = 5.36) coaches, *p* < 0.001, *d* = 1.84, and *p* < 0.001, *d* = 1.72, respectively. The number of images examined did not significantly differ between the Nao and modified Roomba coaches, *p* = 0.64, *d* = 0.15. Similar to the compliance time analysis above, those same two participants had a compliance image score of zero which has been included in this analysis. Results for this analysis can be viewed in Figure 5. Number of images inspected during practice was a statistically significant covariate of compliance images inspected, *F*_(1, 53)_ = 4.88, *p* = 0.03, $\eta_{p}^{2}$ = 0.08, but inclusion of the covariate did not change the pattern of ANOVA results already reported in the paper.

#### 2.2.4. Inspection Time Per Image

**Figure 6:**
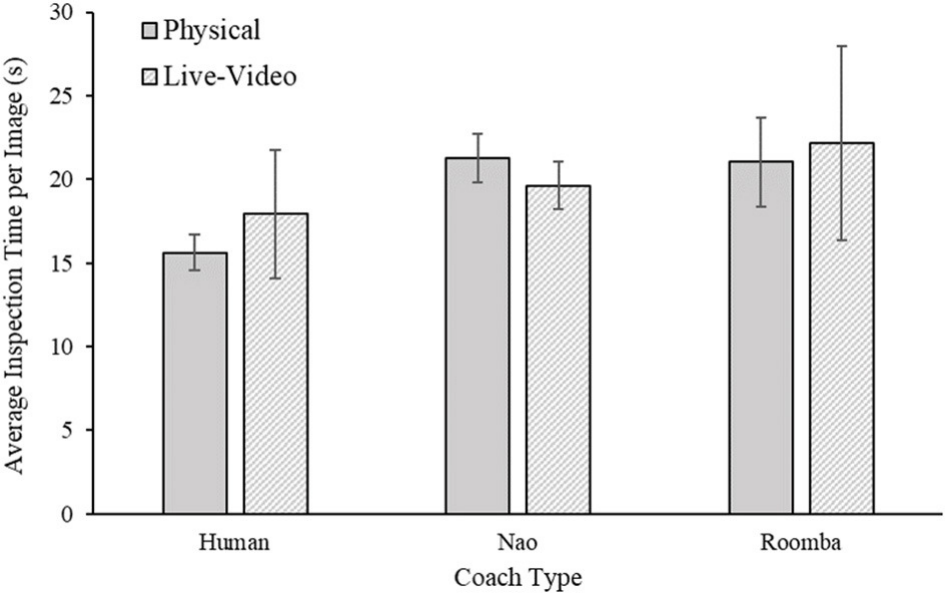
Mean inspection time per image for Study 1. Error bars are standard errors.

As participants complied for a longer duration in the human coach condition, it is perhaps unsurprising that they evaluated more images overall in that condition. However, there is a possibility that participants spent differential amounts of time on average inspecting the SAR images during their compliance period depending on the condition to which they were assigned. Differences across experimental conditions in inspection time could suggest that participants may have worked less diligently at the task in some experimental conditions. To evaluate differences in inspection time per image, a 2 (embodiment) × 3 (coach type) between-subjects ANOVA of mean inspection time per image was calculated. Two participants, one in the physical modified Roomba condition and one in the live-video modified Roomba condition, with a compliance time of zero were excluded from this analysis^1^. The results of the analysis indicated there were no significant main effects of embodiment, *F*_(1, 52)_ = 0.05, *p* = 0.82, $\eta_{p}^{2}$ = 0.00, or coach condition, *F*_(2, 52)_ = 1.35, *p* = 0.27, $\eta_{p}^{2}$ = 0.04, and the embodiment × coach type interaction was also nonsignificant, *F*_(2, 52)_ = 0.22, *p* = 0.80, $\eta_{p}^{2}$ = 0.01. Participants seemed to have spent similar durations, on average, inspecting the SAR images across all experimental conditions. Results for this analysis can be viewed in Figure 6.

#### 2.2.5. Detection Accuracy

**Figure 7:**
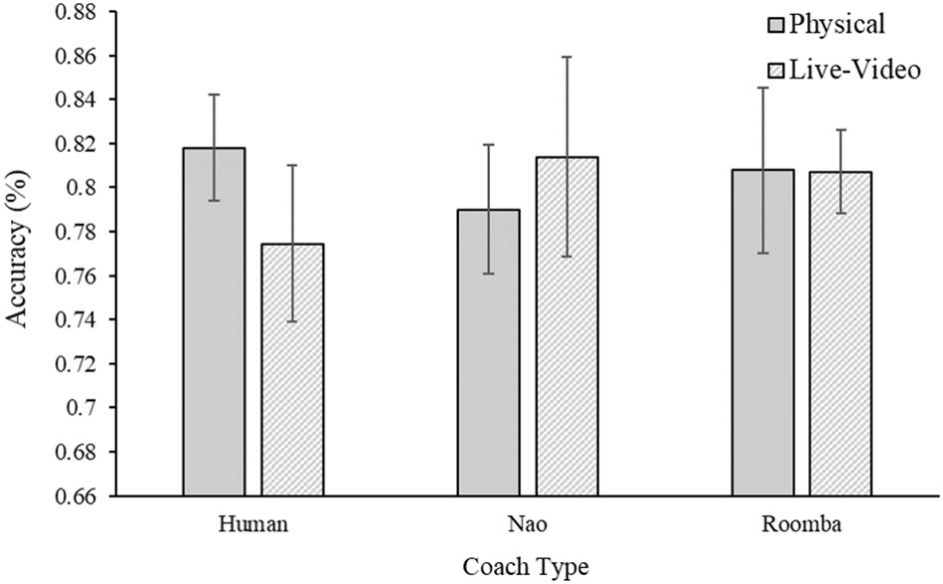
Mean detection accuracy following the first prompt for Study 1. Error bars are standard errors.

As was the case with inspection time per image above, it is possible that participants were differentially accurate in their detections of the targets in each SAR image during their compliance period depending on the experimental condition to which they were assigned. Again, a statistically significant difference in this case may indicate that participants worked less diligently at the task in some experimental conditions. Accuracy was calculated as the percentage of correct target detections in images following compliance to the first prompt. Two participants were excluded from this analysis^1^. A 2 (embodiment) × 3 (coach) between-subjects ANOVA of detection accuracy testing this idea provided no support, as the main effects of embodiment, *F*_(1, 52)_ = 0.00, *p* = 0.80, $\eta_{p}^{2}$ = 0.00, and coach type, *F*_(2, 52)_ = 0.06, *p* = 0.95, $\eta_{p}^{2}$ = 0.00, were nonsignificant. The embodiment × coach type interaction, *F*_(2, 52)_ = 0.55, *p* = 0.58, $\eta_{p}^{2}$ = 0.02, was also not significant. Participants were equally accurate at detecting the targets in the SAR images across experimental conditions. Results for this analysis can be viewed in Figure 7. Inspection time per image in the practice was a statistically significant covariate of inspection time per image during the compliance period, *F*_(1, 53)_ = 15.25, *p* < 0.001, $\eta_{p}^{2}$ = 0.23, but inclusion of the covariate did not change the pattern of ANOVA results already reported in the paper.

#### 2.2.6. Big Five Inventory

Correlations of the five factors of the BFI with our dependent variables is presented in Table 1. As the focus of our research is compliance to a robot coach, the presented correlations were calculated exclusively from participants in the Nao and modified Roomba coach conditions (i.e., they exclude participants from the human coach condition). In addition, as embodiment condition had only a weak effect on compliance, we chose to ignore it (collapse across it) in these analyses. Our results indicated that conscientiousness was positively correlated with the duration of time that participants complied with the robot coach and the number of images they inspected. No other statistically significant correlations between the Big Five and measures of compliance were found.

**Table 1:** Correlations of BFI factors with indices of task compliance for participants in the Nao and modified Roomba coach conditions in Study 1.

**BFI factor**	**Compliance time**	**Compliance images**	**Inspection time per image**	**Detection accuracy**
Agreeableness	0.159	0.083	0.052	0.135
Conscientiousness	0.370^*^	0.329^*^	0.035	0.133
Neuroticism	−0.088	−0.036	−0.144	0.079
Extraversion	0.038	0.107	−0.155	0.151
Openness	0.076	0.132	−0.078	−0.062

* *p < 0.5*.

### 2.3. Discussion Study 1

The purpose of Study 1 was to investigate the effects of embodiment and human-likeness on compliance with a coach. Out of our three compliance measures, only compliance time was affected by embodiment such that being in physical close proximity of the coach resulted in higher compliance times compared to seeing the coach on live video (see Figure 4). This small effect is consistent with findings by Milgram (1974), who demonstrated that requests made in physical proximity elicited greater compliance. It is contrary to our hypothesis, however, because we expected the embodiment condition to have a stronger and consistent effect on compliance in this study. As hypothesized, participants complied significantly more with the human coach than with any of the robots. However, we did not find a significant difference between the two robots. In addition, the effects of human-likeness were not as initially hypothesized—participants complied most to the requests of the human coach, and to a much lesser extent with those of either robot coach. However, comparison of the SAR practice data and participant inspection time and accuracy during their period of compliance to the coach indicated that participants completed the task with similar diligence across conditions, suggesting that differences in compliance were not accompanied by reductions in task directed effort. It is possible that just the appearance of human-likeness between the robots was not sufficient to produce any difference in compliance. The behavior of the robots was identical and because the prompts were made with identical voices it is possible that the attributed mind to the robots were very similar. This finding is consistent with previous work that shows that human-like behavior produces stronger differences for task performance compared to human-like appearance (Abubshait and Wiese, 2017; Fraune, 2020).

Examination of the correlations between the BFI factors and task performance data provided limited support for our initial hypothesis that agreeableness, conscientiousness, and neuroticism would be positively correlated with, and extraversion negatively correlated with, measures of compliance to a robot coach. We found that conscientiousness was positively correlated with the duration of time that participants complied with the robot coach and the number of images they inspected, results that are consistent (in sign and magnitude) with those of Bègue et al. (2015), who found that conscientiousness was positively correlated with compliance in their study. However, we found no statistically significant relationships between our measures of compliance and agreeableness, neuroticism, and extraversion (see Table 1). This pattern of results may suggest that the facets of agreeableness, neuroticism, and extraversion that influence compliance in human-human interactions, such as the desire to preserve social harmony (agreeableness) and avoid conflict (neuroticism), are less activated in human-robot interaction, while facets such as work ethic and rule following, as aspects of conscientiousness, are still important. In other words, the robot coaches employed may not have been perceived as sufficiently human by participants to evoke personality factors associated with interpersonal behavior, but general work ethic remained relevant.

## 3. Study 2

In examining the results of Study 1 with regard to our manipulation of the human-likeness of the robot coaches, it occurred to us that the undifferentiated compliance elicited by the Nao and modified Roomba robots we employed may have been related to the small stature and “cute” appearance of those robots. We considered that a larger robot could potentially be considered more imposing, and thus increase compliance. For example, Fessler et al. (2012) found that the physical size of other humans influences judgments of prospective formidability, and McCluskey et al. (1999) stated that the physical size of an officer would be an important predictor for compliance with a police request. If robot size is to some extent linked to perceived authority and compliance, including a larger robot may result in greater adherence to a robot's request. Additionally, including a large, humanoid robot would address a gap in physical size that is present in many experimental HRI studies, as most commercially available interactive robots are smaller in size than humans.

It is also possible that the level of compliance observed in the sample population of Study 1 may be different than that of a military population, for example, where following orders is instituted through an established chain-of-command. Military officers, particularly those in a chain of command, are expected to provide “a good example of virtue, honor, patriotism, and subordination” (see Snider, 2008). Rosenbloom (2011) found that a military population was more compliant than a civilian population in exhibiting safe road-crossing behaviors. As such, a goal of the second study was to evaluate if such differences in the population also extend to compliance with a robot's request.

Therefore, the aim of Study 2 was to investigate whether members of a special population in which compliance is emphasized, specifically military cadets, would exhibit different patterns of compliance to a coach. Military cadets are of comparable age to the sample population of Study 1, but have been indoctrinated in military culture where compliance is emphasized. Additionally a large humanoid robot, the RethinkRobotics Baxter, was included to address the possibility that the physical size of the robots in Study 1 accounted for the decreased compliance to those coaches. Since embodiment (video vs. physically present) had only modest effects on compliance in Study 1, that manipulation was not included in Study 2. The task paradigm and procedure was otherwise identical to that of Study 1, including inclusion of the BFI as an exploratory measure. A portion of the data used in Study 2 was previously published (Haring et al., 2019). Meta-analyses should only include data presented in this paper and exclude the 2019 publication.

### 3.1. Method

#### 3.1.1. Participants

Ninety-four participants were recruited from the U.S. Air Force Academy (USAFA) participant pool. By the time USAFA cadets are eligible to the participant pool, they have at least completed a rigorous boot camp and initial military training in their first year. The participant pool is mainly freshmen at USAFA, but can contains cadets from all 4 years. Three participants were excluded because of their familiarity with Milgram's research, they suspected the experimental manipulation, or the experimental session was interrupted. As a result, a total of 91 participants (*M*_*age*_ = 18.9, *SD*_*age*_ = 1.07, 39 Females) were included in the final data analysis for Study 2.

#### 3.1.2. Experimental Design

**Figure 8:**
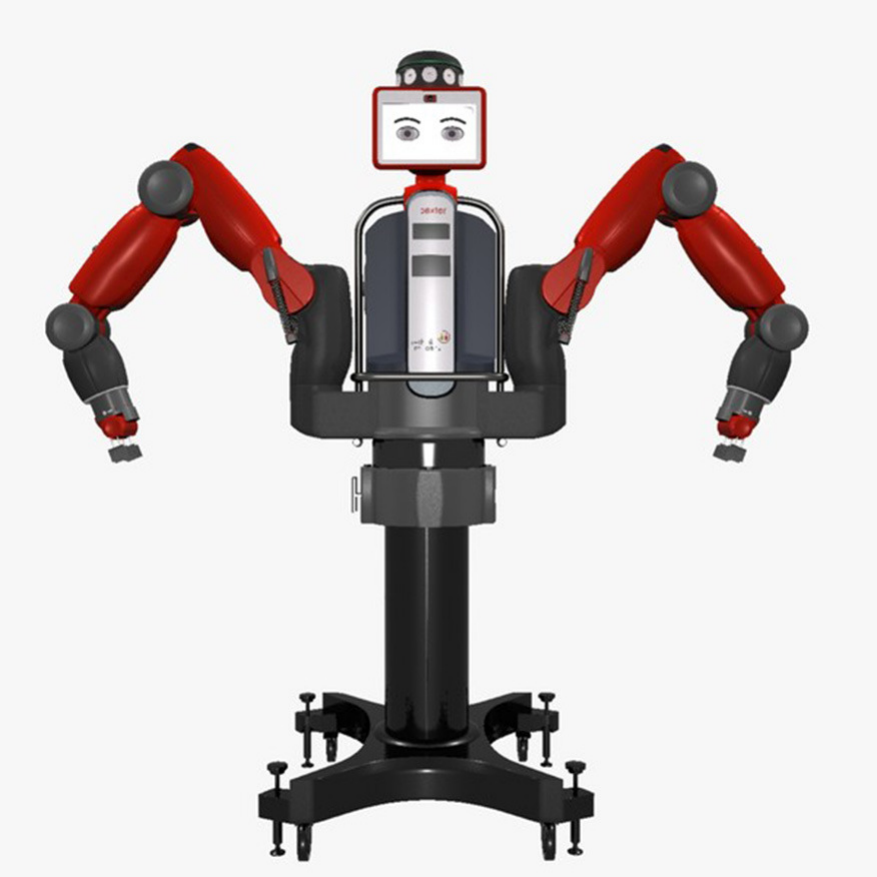
The robot Baxter.

Study 2 employed a one factor between-subjects design with four levels of coach type. The same three coaches used in Study 1 were included: a human (*N*= 20), a Nao robot (*N* = 23), and a modified Roomba robot (*N* = 27). Additionally, a Rethinks Baxter Robot was included as a fourth coach (*N* = 21). As was the case in Study 1, the human coach was always the same person (though not the same person as the human coach from Study 1), a woman, 4th year USAFA Cadet. The Baxter robot was included because it is a large humanoid robot (ABOT score = 27.3). With a height of approximately 6 feet in this study, Baxter is more comparable in size to a human than the Nao and modified Roomba. A picture of the Baxter robot can be seen in Figure 8. In Study 2, all coaches were physically present in the laboratory.

### 3.2. Results

#### 3.2.1. SAR Practice Task

Similar to Study 1, analyses were performed to address the possibility that participants evaluated their need to perform the SAR practice task differentially based on the coach conditions to which they were assigned even before they were exposed to our compliance manipulation. A series of between-subjects ANOVAs were computed comparing the duration in seconds that participants performed the SAR image task from the beginning of the experiment until their first request to advance to the testing phase, the number of SAR images they examined during that period, their mean inspection time per image, and their detection accuracy during that period. On average, participants in all conditions spent approximately the same amount of time, *F*_(3, 87)_ = 1.58, *p* = 0.20, $\eta_{p}^{2}$ = 0.05 (*M* = 524.28 s, *SE* = 0.80 s) examining approximately the same amount of images, *F*_(3, 87)_ = 0.49, *p* = 0.70, $\eta_{p}^{2}$ = 0.02 (*M* = 23.75, *SE* = 0.80) before their first request to move to the testing phase, *F*_(3, 87)_ = 0.28, *p* = 0.84, $\eta_{p}^{2}$ = 0.01 (*M* = 75.33%, *SE* = .80) A one-way ANOVA on inspection time per image before the first prompt did reveal a significant main effect for coach type, *F*_(3, 87)_ = 3.23, *p* = 0.03, $\eta_{p}^{2}$ = 0.10. Participants assigned the Baxter coach (*M* = 18.86 s, *SE* = 0.94 s) inspected images for a shorter duration compared to participants assigned the Nao coach (*M* = 25.88 s, *SE* = 1.83 s).

#### 3.2.2. Compliance Time

**Figure 9:**
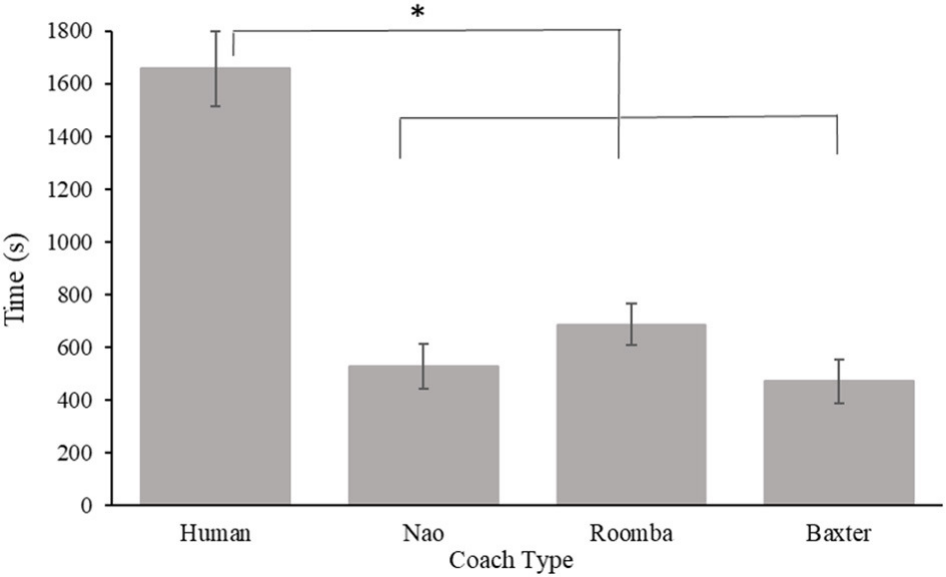
Mean compliance time in Study 2. Error bars are standard errors. The asterisk indicates significant differences.

Similarly to Study 1, compliance time was calculated as the cumulative duration in seconds that participants performed the SAR image task from their first request to advance to the testing phase until the fifth request (after which the task was terminated). A one-way ANOVA of compliance time revealed a significant main effect for coach type, *F*_(3, 87)_ = 31.48, *p* < 0.001, $\eta_{p}^{2}$ = 0.52. *Post-hoc t*-tests with a Bonferroni correction revealed participants complied for a longer duration to a human coach (*M* = 1655.93 s, *SE* = 143.98 s), than to the Nao, *p* < 0.001, *d* = 2.21 (*M* = 527.45 s, *SE* = 84.41 s), modified Roomba, *p* < 0.001, *d* = 1.79 (*M* = 684.94 s, *SE* = 79.91 s), and Baxter, *p* < 0.001, *d* = 2.25 (*M* = 469.17 s, *SE* = 81.65 s) coaches. The total compliance time did not significantly differ between the Nao and modified Roomba coaches, *p* = 0.99, *d* = −0.42, between the Nao and Baxter coaches, *p* = 0.99, *d* = 0.17, or between the modified Roomba and Baxter coaches, *p* = 0.61, *d* = 55. Results for this analysis can be viewed in Figure 9. However, because individual differences in tendency to practice at the task could influence total compliance time, we conducted a follow up analysis of covariance (ANCOVA) using participants' practice time as a covariate in the model. The results of this analysis indicated that participant practice time was not a statistically significant covariate of compliance time, *F*_(1, 86)_ = 3.79, *p* = 0.06, $\eta_{p}^{2}$ = 0.04.

#### 3.2.3. Compliance Images

**Figure 10:**
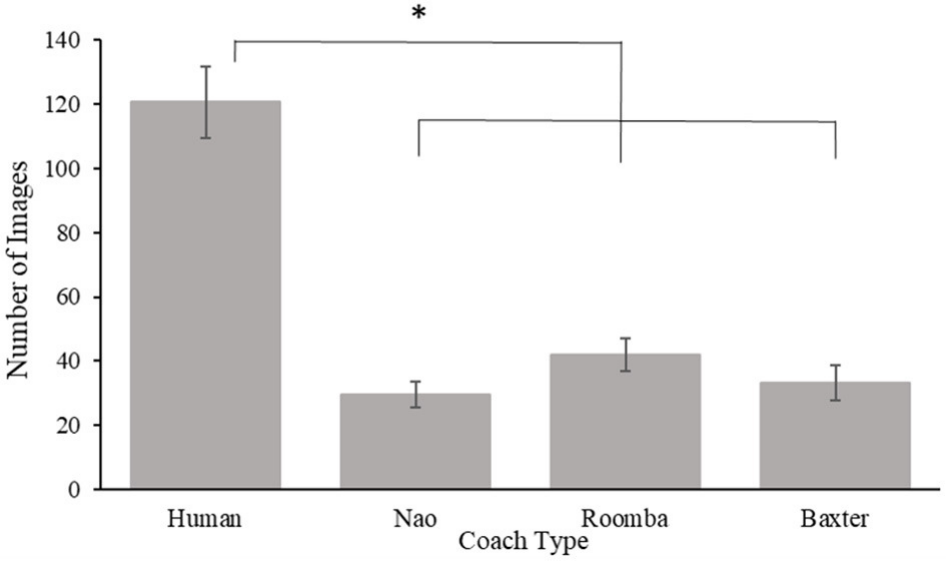
Mean compliance images in Study 2. Error bars are standard errors. The asterisk indicates significant differences.

Compliance was also assessed by the number of SAR images participants inspected following their first request to advance to the testing phase until their fifth request. A one-way ANOVA of the number of images examined revealed a significant main effect for coach type, *F*_(3, 88)_ = 38.97, *p* < 0.001, $\eta_{p}^{2}$ = 0.57. *Post-hoc t*-tests with a Bonferroni correction revealed participants inspected more images with a human coach (*M* = 120.60 images, *SE* = 11.07 images), compared to the Nao, *p* < 0.001, *d* = 2.42 (*M* = 29.48 images, *SE* = 4.07 images), modified Roomba, *p* < 0.001, *d* = 1.98 (*M* = 42.04 images, *SE* = 5.11 images), and Baxter, *p* < 0.001, *d* = 2.22 (*M* = 33.29 images, *SE* = 5.45 images) coaches. The number of images examined did not significantly differ between the Nao and modified Roomba coaches, *p* = 0.97, *d* = 0.54, between the Nao and Baxter coaches, *p* = 0.99, *d* = −0.17, or between the modified Roomba and Baxter coaches, *p* = 0.99, *d* = 0.34. Results for this analysis can be viewed in Figure 10. Number of images inspected during practice was a statistically significant covariate of compliance images inspected, *F*_(1, 86)_ = 6.38, *p* = 0.01, $\eta_{p}^{2}$ = 0.07, but the inclusion of the covariate did not change the pattern of the ANOVA results already reported in the paper.

#### 3.2.4. Inspection Time Per Image

**Figure 11:**
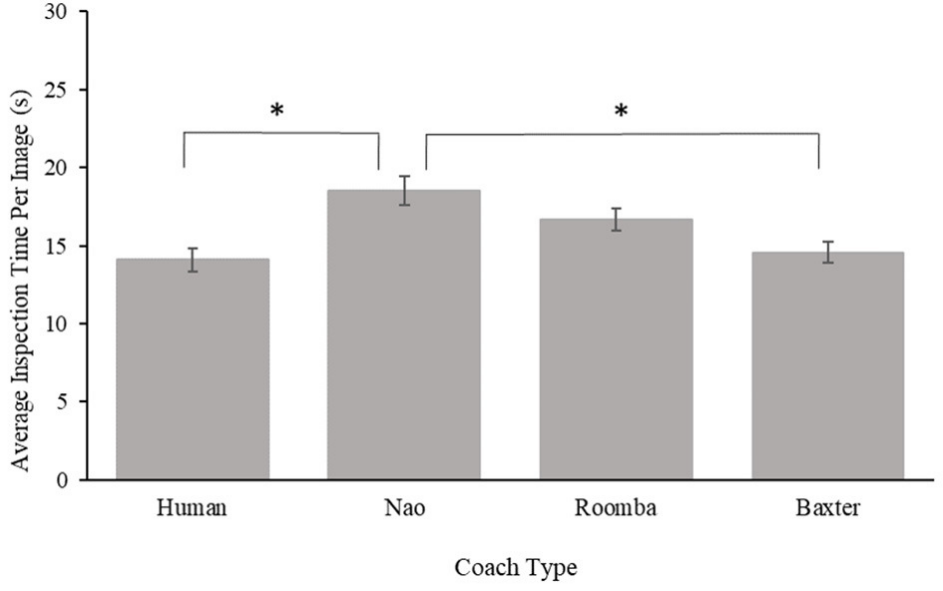
Mean inspection time per image for Study 2. Error bars are standard errors. The asterisk indicates significant differences.

In order to assess the possibility that participants were working less diligently at the task in some experimental conditions, a one-way ANOVA was performed on the mean inspection time per image. Results from this ANOVA revealed a significant main effect for coach type, *F*_(3, 87)_ = 7.75, *p* < 0.001, $\eta_{p}^{2}$ = 0.21. *Post-hoc t*-tests with a Bonferroni correction revealed participants on average inspected images for a longer duration with the Nao (*M* = 18.52 s, *SE* = 0.93 s) compared to the Human, *p* < 0.001, *d* = 1.29 (*M* = 14.11 s, *SE* = 0.74 s) and Baxter, *p* < 0.001, *d* = 1.20 (*M* = 14.57 s, *SE* = 0.67 s) coaches. The mean inspection time did not significantly differ between the Human and Baxter coaches, *p* = 0.99, *d* = 0.14, between the Human and modified Roomba (*M* = 16.69 s, *SE* = 0.69 s) coaches, *p* = 0.07, *d* = 0.75, between the Baxter and modified Roomba coaches, *p* = 0.21, *d* = 0.63, or between the Nao and the modified Roomba, *p* = 0.37, *d* = 0.51. Results for this analysis can be viewed in Figure 11. Inspection time per image in the practice was a statistically significant covariate of inspection time per image during the compliance period, *F*_(1, 86)_ = 10.00, *p* = 0.002, $\eta_{p}^{2}$ = 0.10. The main effect of coach was still significant with the inclusion of the covariate and *post-hoc* comparison results remained the same with the exception of the comparison between the Human and Roomba coaches. Inclusion of the covariate did demonstrate that inspection time per image during the compliance period did differ between the Human and Roomba coaches, but only after individual variance with inspection time per image during practice was accounted for, *p* = 0.02, *d* = 0.86.

#### 3.2.5. Detection Accuracy

**Figure 12:**
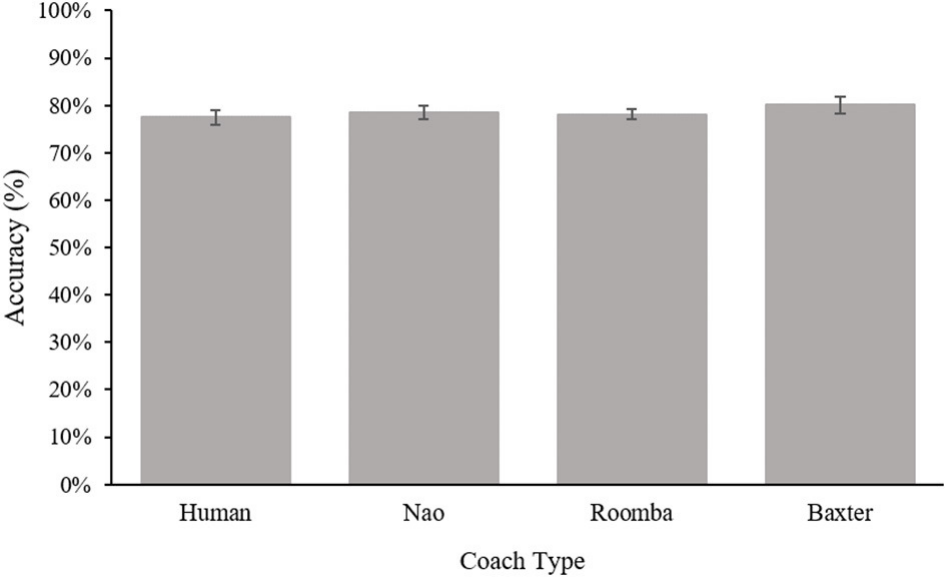
Detection accuracy following the first prompt for Study 2. Error bars are standard errors.

As in Study 1, to assess the possibility that participants worked less diligently at the task in some experimental conditions following their initial request to end the practice phase, a one-way ANOVA was performed on the detection accuracy of inspected images. Accuracy was calculated as the percentage of correct target detection on images following compliance to the first prompt. Results from this ANOVA revealed a nonsignificant main effect for coach type, *F*_(3, 87)_ = 0.65, *p* = 0.54, $\eta_{p}^{2}$ = 0.02. Participants achieved similar target detection accuracy across experimental conditions, *p* = 0.99 for all comparisons. Results for this analysis can be viewed in Figure 12. The accuracy of images inspected during practice was a statistically significant covariate of accuracy during the compliance period, *F*_(1, 86)_ = 11.61, *p* < 0.001, $\eta_{p}^{2}$ = 0.12, but the inclusion of the covariate did not change the pattern of the ANOVA results already reported in the paper.

#### 3.2.6. Big Five Inventory

Correlations of the five factors of the BFI with our dependent variables are presented in Table 2. As was the case in Study 1, the presented correlations were calculated exclusively from participants in the Nao, modified Roomba, and Baxter coach conditions (i.e., they exclude participants from the human coach condition). The results of these analyses indicated no statistically significant correlations between the Big Five factors and measures of compliance. However, there was a not quite significant (*p* < 0.10) effect in the data suggesting that extraversion was negatively correlated with the duration of time that participants complied with the robot coach and the number of images they inspected.

**Table 2:** Correlations of BFI factors with indices of task compliance for participants in the Baxter, Nao, and modified Roomba coach conditions in Study 2.

**BFI factor**	**Compliance time**	**Compliance images**	**Inspection time per image**	**Detection accuracy**
Agreeableness	−0.185	−0.184	0.010	0.076
Conscientiousness	0.026	−0.002	0.076	0.024
Neuroticism	−0.047	−0.016	−0.063	−0.028
Extraversion	−0.201^†^	−0.230^†^	0.085	0.091
Openness	−0.015	0.020	−0.157	0.000

† *p < 0.10*.

### 3.3. Discussion Study 2

The purpose of Study 2 was to further investigate the influence of human-likeness on compliance to a robot coach, including a large robot (Baxter), with a population of military cadets. Our results in Study 2 were generally consistent with those of Study 1, demonstrating that participants complied with requests from the human coach to continue practicing for a longer duration, and inspect more SAR images, than they did for any of the robot coaches employed in this study. Contrary to our initial hypothesis, the large Baxter robot did not elicit greater compliance from participants relative to the Nao or modified Roomba robots, suggesting that robot size, *per se*, is insufficient to elicit greater compliance. Comparison of the SAR practice data and participant inspection time and accuracy during their period of compliance to the coach indicated that participants generally completed the task with similar diligence across conditions, with inspection time per image actually increasing in the Nao condition relative to the human coach condition, suggesting that differences in compliance were not accompanied by reductions in task directed effort.

In addition, again contrary to our initial hypothesis, USAFA cadets displayed a similar degree of compliance to the civilian population employed in Study 1. Though we did not specifically test differences in compliance time across our two studies because of the experimental differences in each, mean compliance time to the human coach (1292.36 s in Study 1 and 1655.93 s in Study 2) and robot coaches (580.99 s in Study 1 and 560.52 s in Study 2) were broadly consistent in each sample, suggesting that military personnel are likely to be no more compliant to a robot possessing a degree of delegated authority than civilians.

Finally, examination of the correlations between the BFI factors and task performance data revealed no statistically significant correlations between them. A trend in the correlation in the data suggested extraversion was negatively correlated with the duration of time that participants complied with the robot coach and the number of images they inspected (see Table 2). This broadly aligns (in both direction and magnitude) with Gudjonsson et al. (2004), who found that extraversion was negatively correlated with a self-reported measure of compliance. However, the statistically significant correlations between conscientiousness and task performance data observed in Study 1 were not evidenced in Study 2. It is not immediately clear why extraversion, and not conscientiousness, would be more relevant in this sample, and it undermines, to some extent, our supposition in Study 1 that participants' reactions to the robot coaches were less driven by social interaction dynamics, and more by work ethic. These outcomes certainly bear further investigation.

## 4. General Discussion

The purpose of our two experiments was to investigate the effects of embodiment and human-likeness on compliance to a robot coach. The combined results from our studies indicate a number of interesting findings. First, the results of Study 1 suggest that embodiment had a fairly weak effect on compliance in our study. Second, compliance was greater to a human coach than to any of the robot coaches we investigated, regardless of the robot's human-likeness or size. However, *less* compliance isn't *noncompliance*—across the two studies, the majority of participants complied with the robot coach's request to continue practicing after they initially indicated their desire to cease. Furthermore, participants continued to diligently perform the task, suggesting they were not superficially invested. Third, compliance was comparable in both the civilian and cadet populations we examined, indicating that people who are experienced at following a hierarchical chain of command appear to be no more compliant to a robot possessing a degree of delegated authority than civilians. Fourth, the effects of personality on compliance were inconsistent across our two studies. Neuroticism was correlated with greater task compliance in Study 1, but not in Study 2, and conscientiousness was correlated with reduced compliance in Study 2, an unexpected outcome given previous research on the trait.

With regard to the fairly weak effects of the robot coaches on participant compliance in our studies, our results broadly align with those of Cormier et al. (2013), who found that participants protested the commands of a robot more frequently and after a shorter task duration than those from a human. It may be that the social cues and norms present in human-human interactions that influence compliance are not activated to the same degree by robots (at least those tested) compared to other humans.

An additional, potential explanation for the observed effects may be that participants did not recognize (or accept) the delegated authority of the coaches in this experiment. Participants were told by the experimenter (the “true” authority figure in this study) that the coaches were provided to assist them in learning the difficult SAR image task (delegated authority from the experimenter). However, several participants complained during the structured debrief interview that they felt the coaches' feedback was not particularly helpful in learning the task. As suggested by Fasola and Matarić (2013) a robot that performs tasks ineffectively (i.e., providing unhelpful feedback during learning) may reduce user's perceptions of the robot's trustworthiness and usefulness in achieving desired goals (learning the SAR image task). This also broadly aligns with research indicating that people are less tolerant of imperfect machines (Madhavan and Wiegmann, 2007; Merritt et al., 2015; Lyons and Guznov, 2019), and the tendency to blame them for failures (Elish, 2019; Hohenstein and Jung, 2020). In our studies, the ineffective feedback may have undermined participants' beliefs in the expertise of the robot coaches at the task, thereby reducing the potency of their delegated authority and the efficacy of their requests to continue performing the task. For the human coaches, however, this effect may have been offset by the social cues and norms activated by the situation.

Nevertheless, although participants exhibited less compliance to the robot coaches employed in this experiment, on average, they still perseverated at the task approximately 10 additional minutes after their initial decision to discontinue practicing. In addition, when they complied, they continued performing the task with the same diligence observed with the human coach. This seems to indicate that, though they may not have felt as pressured to comply with the robot coaches' requests to continue, participants maintained a similar commitment to performing adequately in the SAR task regardless of coach condition, i.e., they were not superficially invested in performing the task with a robot coach while poorly inspecting the images in those conditions.

### 4.1. Personality Factors

With regard to the individual difference factors we investigated, the results were inconsistent across our studies. The correlations we observed in Study 1 between conscientiousness and measures of compliance were comparable to those found by Bègue et al. (2015), leading us to speculate that the personality factors that influence interpersonal dynamics, such as agreeableness and neuroticism, may not have been strongly activated by the robot coaches we employed. However, the results of Study 2 did not align with those of Study 1, and instead we detected a trend correlating extraversion and measures of compliance, similar to those reported by Gudjonsson et al. (2004). It is unclear from this pattern of results what the source of this difference could be, aside from potential differences in the two sample populations that we did not assess. However, our results do suggest that personality factors, such as those of the Five Factor Model (Costa and McCrae, 1992), are likely to influence compliance in human-robot interactions, and suggest that further research regarding this issue is warranted.

### 4.2. Limitations

While Study 2 included a larger humanoid robot comparable in size to a human, the Baxter robot was still rather mechanical in appearance. It would be desirable to include a fully human-looking machine agent, such as a robot like Sophia or Geminoid (ABOT scores of 62 and 92.6, respectively, Haring et al., 2013), or a digital human avatar (De Visser et al., 2016), in future studies. Fully human-looking robots or avatars could activate social norms regarding compliance more strongly, potentially increasing compliance to levels similar to those observed for our human coaches. In this study, all robots used the same synthetic voice as a control. Future studies could consider the effects of adopting varying degrees of human-like speech. It is also noteworthy that Study 1 was conducted with a smaller sample size per condition than Study 2.

In addition, it could be useful in future research to independently manipulate the coach's visual and auditory representations in the task. For example, inclusion of a coach that completely lacks visual representation would allow examination of the effects of a disembodied voice on compliance. Similarly, a condition featuring no visual and auditory representation of a coach, with just the SAR image task, could provide additional information regarding task perseveration in the absence of a supervising coach (although the experimenter will still likely provoke some degree of demand characteristics).

It is possible that the human-likeness for the Roomba was increased somewhat due to the modification of the camera on its top and was seen as having intentional gaze which can affect social attention (Wiese et al., 2012). We did not assess perceived human-likeness within the study. However, if any error was introduced per this mechanism, it was systematic across all conditions. Additionally, we did not find any differences between the different types of robot coaches. If any difference had occurred because of this mechanism (potentially seeing it as more human-like), it does not seem to have meaningfully affected our results.

We also would like to acknowledge that personality as well as intrinsic motivation may exert an important influence on participants' compliance and a tendency to practicing more (Ryan and Deci, 2000). In this study, we decided to address this by including a measure of the Five Factor Model of personality. While the BFI factors of conscientiousness and openness don't completely overlap with intrinsic motivation, they are moderately correlated, and indeed we did find a moderate correlation between conscientiousness and compliance in Study 1. One consideration for future studies on compliance with robots would be to include measure to evaluate the relation of intrinsic motivation and compliance.

Lastly, this study intentionally omitted additional factors that are likely to influence human perception of a robot coach, such as the reliability of the robot's advice, perceived expertise of the robot, and transparency regarding its decision making. Future studies should also explore social-interaction factors such as robot politeness, voice, and gestures. These factors could have additional impact on compliance, and therefore warrant further study.

## 5. Conclusion

Our results, indicating that participants initially engaged the SAR task similarly, achieving comparable performance regardless of coach condition, combined with our results regarding compliance to the robot coaches' requests to continue practice suggest that robots with delegated authority are likely to elicit non-trivial compliance from human teammates in future human-robot interactions. As is the case for compliance to a human, this could be applied in both positive and negative ways.

With regard to positive outcomes, our results support the use of robot coaches for applications such as training. Participants in both studies engaged the SAR image task similarly regardless of coach, indicating that the identity of the coach did not undermine participant commitment to task goals. In addition, participant compliance to the robot coaches was similar in all conditions, suggesting that designers may not need to be overly concerned with issues of human-likeness and embodiment in training settings. Finally, our results suggest that a robot coach may be effective in encouraging trainees to continue practicing a difficult task, even after they have expressed a desire to quit, though not as effectively as a human coach could.

Regarding potential negative outcomes, our results confirm that a degree of compliance to a robot with delegated authority is likely to occur in future human-machine interactions. As compliance does not seem to be affected by factors such as human-likeness and embodiment, designers will need to be aware of this potential. If compliance is undesirable in a situation, compliance defusing strategies, such as reminders that the robot does not have feelings that would be affected by noncompliance, may help avoid those outcomes.

Overall, our results suggest that in future human-robot interactions, humans are likely to be influenced to comply with the suggestions of their robot teammates, though this influence is likely to be weaker than that wielded by their human teammates.

## Data Availability Statement

The raw data supporting the conclusions of this article will be made available by the authors, without undue reservation.

## Ethics Statement

The studies involving human participants were reviewed and approved by Air Force Academy IRB/Air Force IRB. The patients/participants provided their written informed consent to participate in this study.

## Author Contributions

All authors listed have made a substantial, direct and intellectual contribution to the work, and approved it for publication.

## Conflict of Interest

The authors declare that the research was conducted in the absence of any commercial or financial relationships that could be construed as a potential conflict of interest.

## Footnotes

^1^It is worth noting that two participants (one in the physical modified Roomba condition and one in the live-video modified Roomba condition) never complied with the coach's requests, and therefore, had a compliance time of zero. The experimental set-up allowed this as a legitimate response from participants, and therefore, in this analysis and the analysis of the number of images participants inspected following their first request to advance to the testing phase (“Compliance Images,” below), a value of zero was included for those participants. The statistical result patterns did not change when both participants were excluded from analysis.
